# Timing of Magnetic Resonance Imaging (MRI) in Moderate and Severe TBI: A Systematic Review

**DOI:** 10.3390/jcm14124078

**Published:** 2025-06-09

**Authors:** Philipp Geiger, Raphael Gmeiner, Victoria Schön, Ondra Petr, Claudius Thomé, Daniel Pinggera

**Affiliations:** Department for Neurosurgery, Medical University of Innsbruck, 6020 Innsbruck, Austria; philipp.geiger@tirol-kliniken.at (P.G.); raphael.gmeiner@tirol-kliniken.at (R.G.); victoria.schoen@tirol-kliniken.at (V.S.); ondra.petr@tirol-kliniken.at (O.P.); claudius.thome@tirol-kliniken.at (C.T.)

**Keywords:** magnetic resonance imaging, traumatic brain injury, TBI, severe TBI, moderate TBI, pediatric TBI

## Abstract

**Background**: Traumatic brain injury (TBI) remains a significant global health concern with a substantial socioeconomic impact. Although computed tomography (CT) is the primary initial neuroimaging technique, magnetic resonance imaging (MRI) offers a superior detection of subtle brain injuries. However, the ideal timing for MRI in critically ill patients with TBI remains unclear. **Methods**: This systematic literature review focused on the timing and utility of MRI in moderate and severe TBI in the early treatment phase. A comprehensive search was conducted using PubMed, employing specific search terms related to MRI timing and prognostication in TBI. The mean duration from admission to first MRI was examined in the conducting medical center for reference. **Results**: Early MRI, within 72 h post-injury, demonstrated a prognostic value compared with later scans. Diffusion tensor imaging (DTI) performed within 48 to 72 h captured critical pathophysiological changes. The presence of bilateral traumatic axonal injury in the brainstem or thalami on MRI served as a significant predictor of outcome in severe TBI. In pediatric TBI, most institutions performed MRI between seventy-two hours and two weeks post-injury, highlighting variability in practices. The mean interval until the first MRI at the conducting center was 16 days. **Conclusions**: MRI appears to be a valuable tool for prognostication in moderate to severe TBI, offering additional insights beyond those provided by CT. However, the optimal timing and modality for accurate diagnostic and prognostic utility remain uncertain. Current evidence suggests that MRI performed within 72 h after injury in ICU-treated patients with moderate and severe TBI offers valuable prognostic insights compared with delayed MRI, although further research is needed to establish standardized timing protocols and confirm the clinical impact.

## 1. Introduction

Traumatic brain injury (TBI) is a major global health issue with significant public health and socioeconomic consequences, further complicated by its delayed effects. In Germany and Austria, the annual incidence exceeds 300 cases per 100,000 inhabitants [1,2]. In Germany alone, approximately 350,000 patients were hospitalized in 2021 due to TBI, with treatment and long-term costs estimated to be over EUR 2.5 billion. TBI is a leading cause of mortality and lifelong disability in individuals aged 29 to 45, particularly affecting males [3,4]. TBI is graded according to the Glasgow Coma Scale (GCS), with a mild GCS being 15-13, a moderate GCS being 12-9, and a severe GCS being 8-3. Although most cases are classified as mild TBI, about 3% are moderate and 5% are severe. The primary causes include falls, traffic accidents, sports injuries, and workplace accidents. The epidemiological profile of TBI is shifting, with an increasing incidence among older adults due to greater activity and fall-related injuries in an aging population. A 2021 multicenter study from seven German trauma centers found that one in four TBI patients was 75 years or older, with no significant gender difference in this age group [5,6,7,8]. Among children, TBI also represents the primary factor contributing to mortality and impairment [9]. In the United States, the annual impact of pediatric TBI is considerable; emergency departments receive approximately 630,000 yearly visits related to TBI and approximately 7000 children die because of their injuries. The ramifications of severe TBI frequently result in long-term disabilities. Research findings suggest that approximately 60% of affected children require educational support or community-based services within a year of the incident [10]. It is estimated that 145,000 children in the United States are currently coping with disabilities resulting from severe TBI. The economic burden is significant, with annual costs approaching USD 60 billion when extended care and reduced productivity are considered [11]. Accurate long-term neurological prognosis is essential for clinical decision-making. Neuroimaging, particularly magnetic resonance imaging (MRI), seems to be the best tool for assessing TBI [12,13]. CT is the go-to neuroimaging technique for initial TBI assessments, particularly for identifying urgent conditions requiring immediate intervention. However, CT has clear limitations in detecting subtle brain injuries that can impact various aspects of long-term prognosis, such as neurocognitive functioning, functional independence, and return to the activities of daily living, including early-onset ischemia and diffuse axonal injury (DAI). MRI is superior to CT in visualizing early ischemia, DAI, and brainstem damage [14,15,16,17]. This is due to its superior spatial resolution and sensitivity but the ideal timing for undertaking an MRI study of critically ill and comatose patients remains unclear [14,18]. Recent studies indicate that early MRI improves prognostic accuracy for TBI.

Specifically, research on mild TBI has shown that MRI enhances outcome predictions, while specific MRI-based measures of injury severity, such as lesion volume and the presence and extent of microhemorrhages, have been found to correlate with standardized clinical outcomes in both adult and pediatric patients with moderate to severe TBI, as measured by Glasgow Outcome Scale (GOS) scores, functional independence measures, and survival analyses [18,19].

There are numerous MRI sequences available, ranging from conventional structural imaging to more advanced techniques providing metabolic insights. Commonly utilized sequences include T1, T2, T2*, diffusion-weighted imaging (DWI), fluid-attenuated inversion recovery (FLAIR), T2-space, T1 magnetization-prepared rapid acquisition gradient-echo (MPRAGE), susceptibility weighted imaging (SWI), time-of-flight (TOF), pulsed arterial spin labeling (pASL), and proton/phosphorus magnetic resonance spectroscopy (^1^H/^31^P-MRS). These sequences enable a comprehensive assessment of the brain structure, perfusion, and metabolic activity, offering valuable insights into TBI pathology and prognosis. Although advancements in magnetic resonance spectroscopy (MRS) are relatively recent, there are limited data and a lack of standardized recommendations regarding the ideal timing of its use. Additionally, it is still unclear which MRI modality provides the most accurate prognostic insights.

MRI imaging in critically ill TBI patients presents significant logistical and safety challenges compared with CT scanning. Although CT can be completed in minutes, MRI typically requires approximately one hour, during which several complications may arise. Critically ill patients often experience hemodynamic instability, requiring continuous monitoring and rapid intervention capabilities that are limited in the MRI environment. Additionally, these patients may be unable to maintain the prolonged prone-flat position necessary for MRI acquisition due to increased intracranial pressure concerns.

Despite the extensive array of available imaging techniques, the ideal timing for MRI in the assessment of moderate and severe TBI remains uncertain. This review aims to analyze the existing scientific literature to explore the rationale behind MRI timing in critically ill patients and provide recommendations for MRI after TBI.

## 2. Materials and Methods

**Figure 1:**
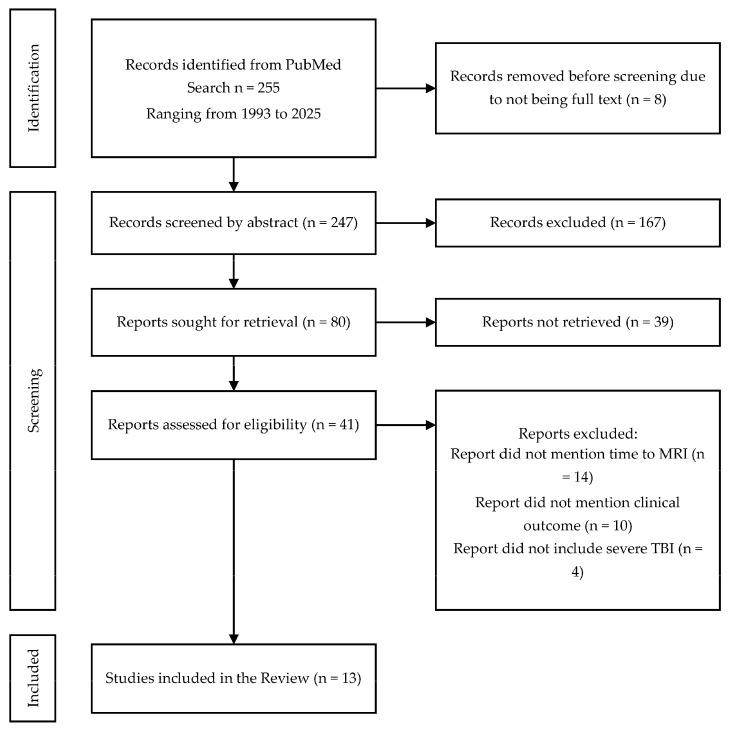
PRISMA chart for inclusion of scientific articles in the review.

A systematic literature review was conducted with the PICO criteria using the search engine www.ncbi.nlm.nih.gov. The search terms used were ((“magnetic resonance imaging”[MeSH] OR “MRI”[tiab] OR “magnetic resonance”[tiab]) AND (“traumatic brain injury”[MeSH] OR “TBI”[tiab] OR “brain trauma”[tiab] OR “head injury”[tiab]) AND (“timing”[tiab] OR “early”[tiab] OR “delayed”[tiab] OR “acute”[tiab] OR “subacute”[tiab]) AND (“moderate”[tiab] OR “severe”[tiab]) AND (“prognosis”[MeSH] OR “outcome”[tiab] OR “predict*”[tiab])). From the search, 255 relevant scientific works from 1993 to 2025 were identified and 13 were deemed relevant (Figure 1). In addition, based on this review, the data regarding MRI timing at our department were analyzed over a period of 2 years. Between January 2023 and January 2025, 15 ventilated patients with severe TBI and who had MRI conducted were identified at our department. The grade of TBI, length of hospital stay, and timing of the first cranial MRI were recorded. The following inclusion criteria for the selected articles were chosen according to the PICO criteria: adult and pediatric patients with moderate and severe TBI, MRI performed at any time point post-injury with timing information reported, different MRI timing groups and CT imaging, prognostic value, prospective and retrospective study designs, and randomized controlled and cohort studies with a minimum of 10 patients.

Using the Newcastle–Ottawa Scale for cohort studies and QUADAS-2 for diagnostic accuracy studies, two independent reviewers evaluated all 13 studies, with disagreements resolved via a consensus discussion with a third reviewer. Common methodological limitations included inadequate control for confounding variables, a lack of blinding in outcome assessments, and variable follow-up periods, all of which informed our interpretation of results and the certainty of our conclusions regarding optimal MRI timing in TBI. The review was not registered.

The analysis of demographic data was achieved using JMP Pro 17 (SAS Inc., Cary, NC, USA). The workflow of the inclusion of scientific works is summarized in Figure 1.

## 3. Results and Discussion

The key aspects of the included scientific works are summarized in Table 1.

### 3.1. MRI in Adult Moderate and Severe TBI

#### 3.1.1. MRI Availability and Use

A discussion of when and if MRI in TBI is a necessity is an avid concern. A survey of Australian clinical professionals on the use of MRI in patients with TBI revealed that 73.4% of respondents ordered MRI scans, while 66% expressed a desire for more frequent utilization [19]. The primary barrier identified was limited access to MRI scanners (57.1%). Additionally, only 42.1% of participants reported conducting advanced MRI analyses, citing a lack of specialized expertise and user-friendly analysis tools as significant challenges. Follow-up MRI in the chronic phase of TBI (>1 month after injury) was only performed in 36% of cases. The severity of TBI ascertained by GCS was the major deciding factor for MRI indication. These findings highlight substantial variability in the purpose, timing, and protocol design of clinical MRI examinations following TBI. Timing varied greatly, with 38% conducting MRI in the acute phase (<24 h) and the vast majority (57.4%) performing a scan in the subacute phase (1 day to 1 month post-injury) [19].

#### 3.1.2. Role of Early Change Detection

A study by Richter et al. [22] based on the CENTER-TBI database revealed that early MRI within 72 h post-injury demonstrated a superior prognostic value compared with later scans. Early MRI could identify critical pathophysiological alterations that influence long-term prognosis. However, the role of MRI in moderate and severe TBI remains unclear, particularly given that patients with mild TBI are typically able to undergo the procedure while awake and at a significantly earlier stage. It is noteworthy that repeated MRI conducted three weeks later revealed an increase in ventricular volume and a decrease in brain volume, which could potentially allow for the prognostication of future sequelae. This suggests the existence of chronic, sustained alterations in the brain tissue after TBI. The researchers demonstrated that MRI conducted within the initial 72 h following the injury yielded more informative biomarkers for outcome estimation than those obtained from scans performed at later stages. This finding is particularly noteworthy in the context of diffusion tensor imaging (DTI) sequences. When performed during the acute phase, typically within 48 to 72 h following the injury, DTI demonstrates an increase in fractional anisotropy (FA) and a concomitant reduction in diffusion capability. It is hypothesized that these changes are the consequence of cytotoxic edema that ensues following the traumatic event. Such early imaging can capture critical pathophysiological changes that may not be as apparent in later scans, potentially offering a more accurate basis for prognosis and treatment planning in TBI cases [22].

In 2018, Humble et al. [21] conducted a study to investigate the correlation between DAI and MRI results within two weeks and the clinical outcome two years later. A regression analysis demonstrated that the severity of the injury, as quantified by the standardized injury severity score, was a significant predictor of the clinical outcome. MRI was performed within two weeks, provided that the patient was able to tolerate the supine position for an adequate period without a significant elevation of intracranial pressure (ICP). The quality of life following the designated follow-up period was evaluated using the Quality of Life after Brain Injury Overall Score (QUOLIBRI-OS). All TBI severity levels, in conjunction with DAI, were included in the study [21].

A 2024 study proposed a grading system for TBI, demonstrating significant findings, particularly in cases of severe TBI. Traumatic axonal injury (TAI) represents a primary type of lesion associated with TBI. The diagnosis of TAI in clinical settings necessitates the utilization of early MRI techniques. Abnormal signals identified in specific regions, predominantly within the white matter, on DWI or FLAIR scans as well as microhemorrhages discerned through T2* gradient-echo (T2*-GRE) or SWI are regarded as biomarkers for TAI. The occurrence of TAI in deeper brain structures is regarded as a more severe phenomenon as it suggests the involvement of great force. The conventional MRI grading system for TAI, developed by Gentry and Adams, classifies injuries into three severity grades. However, its prognostic value remains uncertain [30,31]. In previous studies, the same research team demonstrated that lesions in the midbrain and brainstem on both sides are associated with lower GCS scores. The median time interval between the occurrence of the traumatic event and the initial MRI examination was seven days. Patients were excluded from the study if the required six-week period had elapsed since MRI was performed. Subsequent MRI examinations were conducted at three- and twelve-month intervals following the traumatic event. In cases of severe TBI, the detection of bilateral TAI in the brainstem or thalami serves as a significant predictor of the clinical outcome, particularly when the injury is found in the pons. In contrast, for mild to moderate TBI, the overall volume of contusions observed on MRI is a more significant determinant of the clinical outcome than the volume of TAI [23]. The presence of bilateral lesions in the pons on MRI is significantly associated with an increased risk of mortality [32].

Toth et al. [29] highlighted the temporal evolution of microbleeds in SWI sequences over time. Their cohort received ultra-early MRI (<24 h) after moderate and severe TBI and once again after 1 week. They could show traumatic microbleeds expanding over time, especially in the corpus callosum, coronal radiation, and subcortical white matter. Their conclusion of subsequently decreased microbleeds in number but an increase in volume led to the assumption that localization and volume matter more than quantity [29].

In moderate and severe TBI it may be concluded that timely MRI within the first 72 h may be beneficial for patient prognosis and care as well as long-term treatment. Although the Brain Trauma Foundation (BTF) guidelines primarily focus on CT imaging for the initial assessment, they acknowledge MRI’s superior sensitivity at detecting subtle injuries. Our recommendation for MRI within 72 h complements the BTF’s emphasis on the early detection of secondary injury processes. The ACS Trauma Quality Programs Best Practices Guidelines recognize that MRI has superior sensitivity relative to CT for most acute intracranial findings, and more than 25% of patients with TBI presenting at Level 1 trauma centers with a negative initial head CT are determined to have intracranial injuries upon brain MRI [33].

#### 3.1.3. Magnetic Resonance Spectroscopy (MRS) in TBI

Carpentier and colleagues proposed the use of MRS for the assessment of occult brainstem lesions. The authors proposed that conventional MRI protocols are inadequate for the detection of severe sequelae following severe TBI. A total of 40 patients were included and a single-voxel MRS was conducted on patients with a mean time of 18 days following the initial injury. The Glasgow Outcome Scale (GOS) was evaluated 18 months after the traumatic event. In comatose patients, MRS revealed lesions in the brainstem that were not discernible on conventional MRI. No correlation was identified between MRS disturbances and the anatomical location of MRI lesions. In two patients with a normal MRI, MRS identified severe functional damage to the brainstem (N-acetylaspartate/creatine ratio < 1.5), which was not visible on MRI. These findings were found to have a predictive value only when MRI and MRS were concurrently conducted. When conducted separately, each imaging modality proved to be unable to distinguish between different GOS groups in the long term. Given that the interval between the impact and MRI and MRS scans was up to 18 days, which is beyond the acute unstable period, the longer duration of the MRI/MRS sequences was justified. It can be concluded that these two imaging modalities can and should be used in a complementary manner [20].

### 3.2. Higher Resolution Increases Lesion Detectability

#### 3.2.1. Susceptibility Weighted Imaging (SWI)

In acute settings, CT’s logistical advantages—speed, widespread availability, and compatibility with unstable patients—solidify its role as the first-line imaging modality. However, a study by Lawrence et al. [16] revealed that 46% of moderate to severe TBI patients (GCS ≤ 14) exhibited traumatic cerebral microbleeds (TCMBs) on SWI despite normal or inconclusive CT findings. This discrepancy highlights SWI’s unique capacity to identify microvascular injuries that strongly correlate with initial GCS scores (Spearman’s R = −0.867; *p* < 0.001) and anatomical localization. Such findings position SWI as a vital adjunct in cases where CT underestimates injury severity, particularly in intubated patients with unreliable GCS assessments. A longitudinal analysis demonstrated that reductions in TCMB volume between initial (<24 h) and follow-up (7–15 days) scans paralleled improvements in GCS (R = −0.860; *p* < 0.001), suggesting SWI’s potential to monitor recovery trajectories. Despite these advantages, SWI’s clinical adoption faces barriers. Its longer acquisition time (3–22 min for 3T protocols) limits utility in hemodynamically unstable patients, and manual TCMB quantification introduces measurement variability, especially for submillimeter lesions [16].

#### 3.2.2. Diffusion Tensor Imaging (DTI)

In a retrospective analysis, Asturias and colleagues examined DTI lesions in the corpus callosum and included a total of 440 patients. The mean time interval between the injury and the MRI scan was 100 days. Lesions in the corpus callosum are associated with deep coma and a worse prognosis, particularly in the splenium of the corpus callosum. Nevertheless, the potential for long-term cognitive sequelae associated with these lesions has only been proposed in the context of mild TBI [27].

Shakir and colleagues investigated the prognostic value of DWI. The researchers demonstrated that early (<48 h) MRI with DWI could differentiate between favorable and unfavorable clinical outcomes in adults by examining the ADC values, thus providing a potential tool for prognostication [28].

#### 3.2.3. T2, FLAIR, T2*-GRE, and SWI

The study by Geurts et al. [15] investigated the effectiveness of four MRI sequences—T2-weighted imaging, FLAIR, T2*-GRE, and SWI—at detecting TBI lesions. The findings highlighted that SWI was the most sensitive sequence for detecting small hemorrhagic lesions, identifying significantly more punctate lesions than T2*-GRE, FLAIR, and T2WI. SWI was particularly effective at visualizing lesions in deep brain structures like the brainstem and corpus callosum. In contrast, T2 and FLAIR were less effective at identifying small hemorrhagic lesions but were better suited for visualizing edema and non-hemorrhagic injuries. Inter-rater reliability varied across sequences. For lesion volume, reliability was generally high across all sequences. However, for counting punctate lesions, SWI and T2*-GRE demonstrated excellent reliability. Clinically, the number of punctate lesions detected on SWI and T2*-GRE correlated inversely with initial injury severity, as measured by the GCS, suggesting that these hemorrhagic lesions were meaningful indicators of injury severity. The lesion volume across all sequences showed a weak to moderate inverse correlation with the functional outcomes assessed by the GOS-Extended. The median time to MRI was 6.7 weeks [15].

### 3.3. MRI in Pediatric Moderate and Severe TBI

Ferrazzano et al. [26] conducted a prospective, observational study with the enrollment of 1000 children who met specific criteria, including severe TBI (GCS ≤ 8 after resuscitation), ICP monitoring, and an age range of 0–18 years. The primary outcome measure was the GOS-E Peds at six months post-injury, with supplementary assessments at three months and one year, including neuropsychological testing. This comprehensive approach was designed to evaluate both short-term and long-term outcomes in pediatric TBI patients across multiple centers worldwide. A survey was conducted in 27 institutions, comprising 18 U.S. and 9 international centers. Most institutions (60%) reported conducting MRI examinations between seventy-two hours and two weeks post-injury. Only one center demonstrated consistent adherence to the protocol, performing MRI within the first 24 h. Four other centers typically obtained scans between 24 and 72 h post-injury. In the United States, there was considerable variation in the timing of MRI scans throughout the post-injury period. Nine U.S. centers typically performed MRI within the first week, with four of these obtaining scans within 72 h. The international sites exhibited comparable variability in MRI timing, with scans distributed across a range of post-injury time points. Only one international center adhered to the standard of performing MRI within 72 h of injury [26]. The statistical analysis yielded no statistically significant differences in MRI timing patterns between U.S. and international ADAPT (Approaches and Decisions in Acute Pediatric TBI) study sites. This finding suggests that the observed variability in MRI practices was consistent across the different geographical regions participating in the study. The survey results underscore the significant challenges inherent in the utilization of MRI for clinical studies of TBI. The observed variability in scanning practices and the absence of standardized TBI imaging protocols across participating centers present significant challenges to the effective incorporation of MRI in clinical and research settings.

The prognostic superiority of MRI has been further evidenced in pediatric cohorts, where T2, FLAIR, and SWI sequences distinguished long-term outcomes more effectively than CT [34]. These findings align with metabolic insights from MRS, which revealed reduced NAA/Cr ratios in normally appearing white matter, a biomarker of neuronal dysfunction even in mild to moderate TBI [17]. These metabolic perturbations, invisible to CT, correlate with delayed cognitive deficits, advocating MRI’s integration into acute assessments and follow-up protocols to predict recovery trajectories [17,34].

### 3.4. In-Hospital Safety for Early MRI 

A significant number of trials endeavored to perform MRI scans on patients with moderate and severe TBI at the earliest possible opportunity. This raises the question of whether critically ill patients within the early post-injury period—with hemodynamic instability, intracranial hypertension, continuous infusion therapy, and the need for invasive and non-invasive monitors—truly benefit from early MRI. Our group showed that patient transportation for early MRI after moderate and severe TBI in critically ill patients seemed to be safe. When stable ICP < 25 mmHg was given for at least 4 h before MRI, ICP did not significantly raise during and after MRI. However, it is important to make individual decisions based on the patient’s status [35,36].

### 3.5. MRI Timing at the Medical University, Innsbruck

Inspired by the present review, we also analyzed the data from our own center, where we found a considerable variability in the timing of the first MRI scan for severe TBI patients. The wide range of 1 to 60 days, coupled with a standard deviation of 14.51 days, indicates the problems of timely imaging for prognosis estimation. This variability raises questions about the factors influencing MRI scheduling and potential implications for patients’ clinical outcomes. The right-skewed distribution of MRI timing was evident from the discrepancy between the mean (15.53 days) and median (13 days). This wide spread prompted considerations of the clinical decision-making processes and resource-allocation strategies in place. Early imaging could be crucial for clinical decision-making, while delayed scans could be due to patient instability or resource constraints. However, the extended delays seen in some patients raise concerns about potential missed opportunities for early intervention or the adjustment of treatment strategies, like more aggressive ICP management protocols or the optimization of cerebral perfusion pressure. The patient characteristics are summarized in Table 2.

## 4. Limitations

This systematic review was limited by significant heterogeneity among the included studies regarding imaging protocols, MRI sequences, and the timing of acquisition, which restricted direct comparisons and the generalizability of findings. The variability in patient populations across studies, including differences in age, injury mechanisms, and TBI severity classification methods, further complicated the interpretation of the results. Outcome measures and evaluation timepoints were inconsistently applied, limiting the comparability of prognostic conclusions. Clinical confounding factors such as sedation protocols, medication effects (including vasopressors, anticonvulsants, and osmotic agents), and targeted temperature management were inadequately controlled for or reported across studies. The practical implementation of early MRI faces real-world challenges, as evidenced by the significant variability in MRI timing at our own institution (1–60 days), highlighting constraints related to patient stability, resource availability, and institutional protocols.

## 5. Conclusions

In conclusion, this comprehensive review of the literature underscores the critical role of MRI in the assessment and prognostication of moderate and severe TBI. The timing of MRI acquisition emerges as a crucial factor for maximizing its diagnostic and prognostic utility. Current evidence suggests that early MRI, particularly within the first 72 h post-injury, offers valuable prognostic insights compared with delayed scans, especially in detecting diffuse axonal injury and early ischemic changes that may be missed on CT imaging. For pediatric patients with moderate and severe TBI, the evidence for optimal MRI timing is particularly limited. Although MRI clearly adds a prognostic value for this population, the significant variability in practice patterns across institutions underscores the need for dedicated pediatric studies to establish age-appropriate timing guidelines that account for developmental considerations and the unique pathophysiology of pediatric TBI. However, the optimal timing and specific MRI modalities for accurate prognostication remain uncertain. Although our findings support the utility of early MRI in ICU-treated patients with moderate and severe TBI, significant variability in clinical practice exists, as demonstrated by both the literature and our institutional experience. The implementation of standardized imaging protocols, timing guidelines, and safety procedures for critically ill patients requires further research to establish best practices and confirm the clinical impact. Future studies should focus on determining the most effective MRI sequences and developing consistent approaches to maximize the prognostic value of MRI in TBI management.

## Figures and Tables

**Table 1:** Table of scientific articles included in the literature review and analysis.

	Author	Year	Type of Article	Patients Included	Time to MRI	TBI Grade
1	Carpentier et al. [20]	2006	Scientific Article	40	Day 17.5 (6.4)	Severe
2	Humble et al. [21]	2018	Scientific Article	311	In the subacute phase (within 2 weeks after injury)	Mild, moderate, and severe
3	Richter et al. [22]	2022	Scientific Article	65	Subacute phase (<30 days)	Moderate and severe
4	Moen et al. [23]	2024	Scientific Article	463	Within the acute phase (1 week)	Mild, moderate, and severe
5	Haghbayan et al. [24]	2017	Review	N/A	Median time < 29 days	Moderate and severe
6	Potapov et al. [25]	2014	Scientific Article	278	Within 21 days	Moderate and severe
7	Ferrazzano et al. [26]	2024	Scientific Article	1000 pediatric patients	Within 30 days	Severe
8	Asturias et al. [27]	2023	Scientific Article	446	<100 days	Mild
9	Shakir et al. [28]	2016	Scientific Article	76	<48 h	Moderate and severe
10	Caeyenberghs et al. [19]	2025	Questionnaire Study	81 respondents	<1 month	Mild, moderate, and severe
11	Toth et al. [29]	2016	Scientific Article	5	<24 h	Moderate and severe
12	Lawrence et al. [16]	2017	Scientific Article	13 patients; 10 healthy controls	<24 h	Mild, moderate, and severe
13	Geurts et al. [15]	2012	Scientific Article	56	6.7 weeks	Mild, moderate, and severe

**Table 2:** Demographics of analyzed institutional patients. Outcome at discharge was measured via the modified Rankin scale (mRS).

Patient Number	TBI Grade	Age	Days to MRI	Outcome at Discharge (mRS)
1	3	21	25	1
2	3	44	10	4
3	3	58	13	3
4	3	67	12	6
5	3	23	11	0
6	3	25	29	4
7	3	79	60	4
8	3	50	13	3
9	3	58	13	2
10	3	33	1	0
11	3	53	2	5
12	3	58	8	2
13	3	71	18	3
14	3	59	15	2
15	3	25	3	3

## Data Availability

All data is provided via tables in the text. The included articles are available via PubMed.
